# In Pursuit of COVID-19 Treatment Strategies: Are We Triggering Antimicrobial Resistance?

**DOI:** 10.1017/dmp.2020.492

**Published:** 2020-12-22

**Authors:** Furqan Khurshid Hashmi, Naveel Atif, Usman Rashid Malik, Fahad Saleem, Zineb Riboua, Mohamed Azmi Hassali, Muhammad Hammad Butt, Tauqeer Hussain Mallhi, Yusra Habib Khan

**Affiliations:** 1 Punjab University College of Pharmacy, University of the Punjab, Allama Iqbal Campus, Lahore, Pakistan; 2 Center of Drug Safety and Policy Research, School of Pharmacy, Xi’an Jiaotong University, Xi’an, Shaanxi, China; 3 Faculty of Pharmacy, University of Balochistan, Quetta, Balochistan, Pakistan; 4 McCourt School of Public Policy, Georgetown University, Washington DC, USA; 5 School of Pharmaceutical Sciences, Universiti Sains Malaysia, Penang, Malaysia; 6 Faculty of Pharmacy, University of Central Punjab, Lahore, Punjab, Pakistan; 7 Department of Clinical Pharmacy, College of Pharmacy, Jouf University, Kingdom of Saudi Arabia

Coronavirus disease 2019 (COVID-19) poses substantial challenges to social life and health-care systems across the world. Because there is no specific treatment against severe acute respiratory syndrome coronavirus 2 (SARS-CoV-2), repurposing of antimicrobial agents remains a mainstay in managing COVID-19 patients. However, the expanded use of broad-spectrum antibiotics carries significant risks of antimicrobial resistance while managing COVID-19 patients. In this context, the World Health Organization (WHO) has recommended preventive and precautionary measures, such as social distancing, self-isolation, hand hygiene, and lockdowns, as effective disease containment modalities during the pandemic.^1,2^

Existing data demonstrates that COVID-19 is associated with secondary infections, such as pulmonary pneumonia and ventilator-associated pneumonia.^3^ In the case of secondary infections, use of antimicrobials is the only treatment strategy left for physicians, especially for suspected bacterial infections.^4^ The crisis of antimicrobial resistance (AMR) is becoming devastating with each passing year, which has cautioned health-care providers to be judicious with the use of antimicrobial agents.^5,6^ The current COVID-19 pandemic threatens to further jeopardize the use of available antimicrobial agents as treatment strategies.

Following the H1N1 influenza outbreak in 2009, several environmental microbiologists warned that the excessive use of antibiotics might lead to an increase in resistant bacterial infections, while during the current COVID-19 pandemic, many cases were treated excessively with antibiotics.^3^ The majority of COVID-19 patients are either asymptomatic or may experience mild to moderate illness without bacterial infection.^7^ However, uncertainty among health-care professionals about the prudent use of antibiotics among COVID-19 patients is an intimidating factor in terms of additional health-care costs, especially for countries with limited resources and poor quality of health services.^8^ Unnecessary antibiotic use in viral diseases and epidemics is leading to an increased AMR and, thus, has amplified the financial burden.^9^ Because AMR has already been declared as a global emergency, health-care professionals need to be careful with the use of antimicrobial agents for the treatment of secondary infections that may arise during the course of COVID-19 and other viral diseases.

Implementation of antimicrobial stewardship programs (ASPs) has been associated with decreased antibiotic use and reduced costs,^10^ which ultimately reduces the AMR burden. WHO’s interim guidelines on the clinical management of COVID-19 suggest the integration of ASPs in the health-care system. These guidelines have discouraged the use of antibiotics in mild or moderate COVID-19 patients without symptoms of a bacterial infection. However, antibiotics classified in WHO’s AWaRe (access, watch, reserve) category, such as co-amoxicillin, could preferably be used in geriatrics and pediatrics.^11^

Health-care authorities and professionals should take hard lines to implement and practice the WHO’s guidelines during management of COVID-19 patients.^12^ Prolonged stay of patients in hospitals resulting from secondary and super-infections may further put unnecessary strain on health-care systems. A multipronged approach using evidence-based practices should be considered for treatment of COVID-19 patients. In addition, compliance with the WHO’s Global Action Plan on AMR is of utmost importance. This can help reduce the additional health-care costs and burden on overwhelmed health-care systems.

Periodic sensitivity testing will help to ascertain the pattern of prevailing AMR so that timely and effective combat strategies can be initiated. Pharmacists and other health-care professionals can play a pivotal role in implementation of ASPs to reduce the injudicious use of antimicrobials during the current COVID-19 pandemic. In a nutshell, appropriate ASP implementation and monitoring can help allocate resources to manage global public health crises effectually.
